# Non‐24‐hour sleep‐wake disorder successfully treated with the combination of ramelteon and suvorexant in a case of autism spectrum disorder

**DOI:** 10.1002/npr2.12142

**Published:** 2020-09-29

**Authors:** Masaaki Iwata, Koichi Kaneko

**Affiliations:** ^1^ Department of Neuropsychiatry Faculty of Medicine Tottori University Yonago Japan

**Keywords:** autism, melatonin, non‐24 hour sleep‐wake disorder, ramelteon, suvorexant

## Abstract

**Introduction:**

Non‐24‐hour sleep‐wake disorder (N24SWD) is often observed in the visually impaired and those who isolate indoors. Melatonin receptor agonists may be used for treatment, but there is currently no evidence that they are effective in patients without visual impairment.

**Case:**

We report a case of a 23‐year‐old woman who withdrew from her social life owing to autism spectrum disorder and experienced an unusual sleep rhythm. She presented with N24SWD. The N24SWD cycle averaged 25.6 days but was extended to 42 days using ramelteon. However, this was not enough. We prescribed the addition of suvorexant and the sleep cycle returned to normal.

**Conclusion:**

N24SWD is a disease that seriously impairs social life and productivity. We propose a possible treatment strategy for N24SWD using ramelteon and suvorexant.

## Abstract 2

We present a case in which the combination therapy of ramelteon and suvorexant was effective for a patient with autism spectrum disorder with non‐24‐hour sleep‐wake disorder (N24SWD) owing to social withdrawal. Here, we discussed the pharmacological mechanism of the combination of ramelteon and suvorexant as a possible therapeutic strategy for patients with N24SWD.

## Graphical abstract

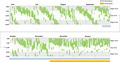

## 1 INTRODUCTION

Non‐24‐hour sleep‐wake disorder (N24SWD) is a relatively rare disorder that is often observed in the visually impaired and those who isolate indoors.^1^, ^2^, ^3^ In addition to lifestyle guidance and high‐intensity light therapy, the use of vitamin B12 and sleeping pills is recommended for treatment.^1^, ^2^, ^4^, ^5^, ^6^ However, with regard to hypnotics, benzodiazepine hypnotics have almost no effect on human biological rhythms themselves; thus, a therapeutic effect of a benzodiazepine hypnotic alone cannot be expected.^7^ Ramelteon, a melatonin receptor agonist, reverses a shifted sleep phase back to normal.^8^ However, the usefulness of ramelteon in N24SWD has not been shown so far. N24SWD is a disease that significantly impairs the patient's quality of life,^2^ and it is expected that a treatment method will be established in the future. Here, we report a case of autism spectrum disorder (ASD) with N24SWD successfully treated with a combination of ramelteon and suvorexant.

## 2 CASE

A 23‐year‐old sighted woman visited our hospital seeking treatment for a delayed sleep phase disorder that progressed each day.

**Figure 1:**
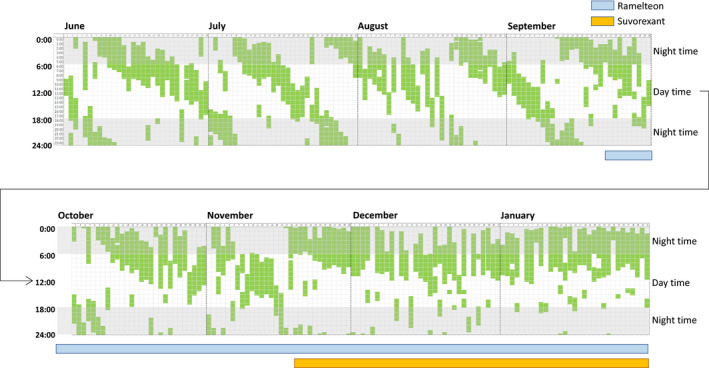
In September, we initiated behavioral education but, owing to a lack of improvement, we prescribed ramelteon 8 mg from the end of September. Although the N24SWD cycle was extended from 25.6 to 42 d, the patient did not completely recover. Thus, we added 15 mg of suvorexant, and the sleep rhythm was restored

Her development was not typical. Since early childhood, although her mother darkened the room to put her to sleep, she was active and found it difficult to sleep at night. She had difficulty getting up in the morning but was able to attend school. When her mother called her, she sometimes gave no reply; thus, the mother initially thought she was deaf, but she was not. She covered her ears when watching TV, which might mean hypersensitivity. Because she tried to stick to her own interpretations and methods, her mother thought of her as a difficult child to raise. Since she was a child, she tended to repeat the same play endlessly and liked reading books rather than playing with her friends. She realized that she did not get along well with her friends but tried to think that "I have to get along with my friends well" and tried to integrate. However, in junior high school, she was left out of her group of friends and was unable to attend school. There was no noticeable bullying but she later attended a special classroom, after which she went to a correspondence high school. She entered college, left her parents, and lived with her sister. However, she often felt anxious, calling her mother with a pessimistic view of life and said that "I hate living." She was so embarrassed to write her thoughts on a class report that she could not submit it and dropped out of college. Since then, she has lived at her parents’ home, sometimes helping with housework. After dropping out of college, her sleeping hours became different day by day and her circadian rhythm was often completely reversed. Initially, she was sleepy but could not sleep for approximately 2 hours when she went to bed. The sleep cycle is now delayed by one cycle every 20 to 30 days (approximately every 25 days). She tried to control herself for 3 to 4 years but could not do so; therefore, she visited our hospital. She brought her sleep records to date, which show that she had six of these delayed cycles in 154 days; thus, her sleep rhythm averaged one of these cycles every 25.6 days (Figure 1).

She was diagnosed with ASD using the *Diagnostic and Statistical Manual of Mental Disorders* version 5, characterized by persistent challenges with social communication and social interaction, and by the presence of restricted, repetitive patterns of behavior, interests, or activities. Additionally, using International Classification of Sleep Disorders (ICSD) criteria, she was diagnosed with N24SWD. She had no other notable physical illnesses.

She received behavioral education, but her sleep rhythm did not improve. Then, we initiated treatment with 8 mg of ramelteon every night around 11:00 PM, and the N24SWD cycle was extended from 25.6 days to 42 days, but did not completely recover. Therefore, we added 15 mg of suvorexant, and the sleep rhythm was restored (Figure 1). Subsequently, she began to take steps to go outside and can now go to a social rehabilitation facility once or twice a week.

## 3 DISCUSSION

We herein report a case of a patient with ASD who suffered from N24SWD. To our knowledge, this is the first report of successful treatment with a combination of ramelteon and suvorexant to a sighted patient with ASD with N24SWD.

ASD is a neurodevelopmental disorder characterized by impairments in communication skills and social interaction, as well as restricted, repetitive, and stereotyped patterns of behavior.^9^, ^10^ Among the most common comorbidities in ASD, sleep disorders^11^ occur in 40% to 80% of cases, which exceeds the 20% to 40% in children with typical development. The presence of sleep disorders in individuals with ASD may exacerbate associated behavioral disorders and lead to the intensification of existing autistic symptoms.^12^, ^13^ Among the etiological hypotheses, atypical production of melatonin may explain the high prevalence of sleep disorders in ASD.^9^, ^10^, ^14^, ^15^

Our case was a patient with ASD who displayed more severe symptoms after dropping out of college. When she was at school, she managed to maintain a standard sleep cycle but N24SWD became apparent once she stopped going outside.

N24SWD is a relatively rare condition that is characterized by a chronic steady pattern of approximately 1‐hour delays in spontaneous sleep‐onset and wake times in individuals living under normal environmental conditions.^16^ For example, for a patient who is delayed by 1 hour every day, the day for the patient is 24 hours + 1 hour = 25 hours. It is difficult to fall asleep at a certain time and wake up in the morning. Eventually, a complete day‐night reversal occurs; thus, approximately every month, social life is disturbed owing to insomnia at night and excessive sleepiness during the day.^2^ Patients who try to maintain a constant sleeping time often complain of periodic insomnia and difficulty waking. If a patient wakes up during day sleep hours, they may experience drowsiness, decreased attention, difficulty in maintaining concentration, fatigue, and malaise.^2^ A diagnosis can be achieved by recording sleep diaries and clarifying sleep/wake patterns.^2^

N24SWD is defined according to four ICSD‐3 criteria: (a) history of insomnia, excessive daytime sleepiness, or both, which alternate with asymptomatic episodes, owing to misalignment between the 24‐hour light‐dark cycle and the nonentrained endogenous circadian rhythm of sleep‐wake propensity; (b) symptoms persist over the course of at least 3 months; (c) daily sleep logs and actigraphy for at least 14 days, preferably longer for blind persons, demonstrate a pattern of sleep and wake times that typically delay each day, with a circadian period that is usually longer than 24 hours; and (d) the disorder is not better explained by another current sleep disorder, medical or neurological disorder, mental disorder, and substance use disorder.^2^, ^17^

At least 50% of totally blind persons (ie, those with no light perception) are thought to suffer from the disorder.^18^ Although the etiology in the blind is a loss of photic input to the pacemaker,^19^ the pathophysiology among sighted individuals is unknown.^7^

No visual impairment was observed in our case, but the symptoms of N24SWD became apparent after the patient dropped out of college and stayed inside the home. In a report by Watanabe et al, the symptoms of N24SWD also became apparent owing to a major life event, in which the patient quit his job in search of a new one.^20^

There are few effective treatments for N24SWD. As benzodiazepine hypnotics have almost no effect on human biological rhythms themselves, they cannot be expected to be effective alone in the treatment of N24SWD ^7^; however, they may be effective when used in combination with high‐intensity phototherapy. The administration of melatonin may be effective for N24SWD in blind but not sighted patients. The only drug that is indicated for N24SWD is tasimelteon, a selective agonist of the melatonin receptors MT_1_ and MT_2_, which can be prescribed in the United States and Europe.^21^, ^22^

Ramelteon is also a sleep agent that selectively binds to the MT_1_ and MT_2_ receptors.^8^ Ramelteon is efficacious in the “treatment of insomnia characterized by difficulty with sleep onset” ^8^ but, as ramelteon is an agonist of the melatonin receptor that controls sleep rhythm, it is expected to have an effect on sleep rhythm disorders.

Melatonin agonists, such as ramelteon, are thought to act on the circadian clock in the suprachiasmatic nucleus of the hypothalamus via the activation of endogenous melatonin receptors.^3^ In our case, the oral administration of ramelteon improved the circadian rhythm but its effect was insufficient. However, with the addition of suvorexant, it is possible to secure a sleep rhythm to the extent that daily life can be carried out and social participation becomes possible.

Suvorexant is an orexin receptor antagonist being developed for the treatment of insomnia.^8^, ^23^, ^24^ Extensive research has demonstrated that the orexin system plays a critical role in the regulation of the transition between sleep and arousal, especially inducing wakefulness.^8^, ^23^, ^25^ Therefore, suvorexant may have improved sleep‐wake rhythm by suppressing the part of wakefulness.

Although it has already been reported that ramelteon can improve N24SWD,^20^ to the best of our knowledge, there are not many reports of the therapeutic effects of ramelteon on N24SWD and no randomized controlled or large clinical trials. In our case, ramelteon extended her N24SWD cycle but not by enough. Further, we have shown that suvorexant may be an adjunctive treatment to improve sleep cycles with ramelteon.

Thus far, the effectiveness of suvorexant against N24SWD has not been reported; however, it remains possible that suvorexant monotherapy is effective against N24SWD.

A recent paper found that the orexin nerves express MT_1_ receptors but not MT_2_ receptors in the perifornical lateral hypothalamus. It was also suggested that melatonin inhibits orexin neurons via MT_1_ receptors, thereby promoting sleep.^26^ Thus, suvorexant, an orexin receptor antagonist, enhances the effect of ramelteon on MT_1_ stimulation. Among the melatonin receptors, the stimulation of MT1 receptors causes the suppression of nerve firing, whereas the stimulation of MT_2_ receptors is known to have a sleep phase‐advancing effect.^27^, ^28^ As suvorexant does not appear to be associated with MT_2_ action, sleep phase advance is not expected; thus, the effect of suvorexant monotherapy on N24SWD remains questionable.

In our case, the effect of ramelteon on circadian rhythm modification was observed, as evident from the extension in the N24SWD cycle from 25.6 days to 42 days following ramelteon administration. Pharmacologically, this is mainly attributed to ramelteon; however, in our case, the sleep phase‐advancing effect of ramelteon and the neuroleptic suppressive effect of suvorexant might have acted synergistically. Further research is expected in the future.

## 4 CONCLUSION

We present a case in which the combination therapy of ramelteon and suvorexant was effective for a patient with ASD with N24SWD owing to social withdrawal. Ramelteon exhibits an agonistic effect on melatonin MT_1_ and MT_2_ receptors, as does tasimelteon; therefore, ramelteon may be as effective as tasimelteon, which has an effect on N24SWD. However, melatonin receptor agonists have not been fully demonstrated to be effective in patients without visual impairment. Based on our results, we propose a combination of ramelteon and suvorexant as a possible therapeutic strategy for sighted patients with N24SWD.

## CONFLICT OF INTEREST

M. I. received honoraria as a speaker from MSD KK KK declares no conflict of interest.

## AUTHOR CONTRIBUTIONS

M. I. treated the patient and drafted the manuscript. K. K. critically reviewed the draft and revised it. All authors made substantial contributions, drafted the manuscript, and approved the final manuscript.

## INFORMED CONSENT

Informed consent was obtained from the patient.

## Data Availability

Data sharing not applicable to this article as no datasets were generated or analyzed during the current study.
